# The LEGACy study: a European and Latin American consortium to identify risk factors and molecular phenotypes in gastric cancer to improve prevention strategies and personalized clinical decision making globally

**DOI:** 10.1186/s12885-022-09689-9

**Published:** 2022-06-13

**Authors:** Tessa Suzanne van Schooten, Sarah Derks, Elena Jiménez-Martí, Fatima Carneiro, Ceu Figueiredo, Erika Ruiz, Maria Alsina, Cristina Molero, Marcelo Garrido, Arnoldo Riquelme, Carmelo Caballero, Eva Lezcano, Juan Manuel O’Connor, Federico Esteso, Judith Farrés, José Manuel Mas, Florian Lordick, Jeannette Vogt, Antonella Cardone, Charis Girvalaki, Andrés Cervantes, Tania Fleitas, on behalf of the members of LEGACy consortium

**Affiliations:** 1 Amsterdam UMC-location VUMC, Department of Medical Oncology, Cancer Center Amsterdam, Amsterdam, The Netherlands; 2 Oncode Institute, Utrecht, The Netherlands; 3 Instituto Investigación Sanitaria INCLIVA (INCLIVA), CIBERONC, Medical Oncology Department, Hospital Clínico Universitario de Valencia, Universitat de Valencia, Valencia, Spain; 4 Institute of Pathology and Molecular Immunology of the University of Porto (IPATIMUP)/Institute of Research and Innovation in Health (i3S); Faculty of Medicine, University of Porto, Porto, Portugal; 5 Instituto Nacional de Cancerología (INCAN), Translational Medicine Laboratory & GI Cancer Department, Mexico City, Mexico; 6 Valld’Hebron Institute of Oncology (VHIO), Medical Oncology Department, Barcelona, Spain; 7 Pontificia Universidad Católica de Chile (PUC), Department of Hemato-Oncology, Santiago, Chile; 8 GenPat, Asunción, Paraguay; 9 Instituto de Previsión Social, Asunción, Paraguay; 10 Instituto Alexander Fleming (IAF), Medical Oncology Department, Buenos Aires, Argentina; 11 Anaxomics Biotech, S.L. (ANAX), Barcelona, Spain; 12 Universitaet Leipzig (ULEI), Medical Oncology Department, Leipzig, Germany; 13 European Cancer Patient Coalition (ECPC), Brussels, Belgium

**Keywords:** Gastric cancer, Tumor microenvironment, Prevention

## Abstract

**Background:**

Gastric Cancer (GC) is the fourth most deadly cancer worldwide. Enhanced understanding of its key epidemiological and molecular drivers is urgently needed to lower the incidence and improve outcomes. Furthermore, tumor biology in European (EU) and Latin American (LATAM) countries is understudied. The LEGACy study is a Horizon 2020 funded multi-institutional research approach to 1) detail the epidemiological features including risk factors of GC in current time and 2) develop cost-effective methods to identify and integrate biological biomarkers needed to guide diagnostic and therapeutic approaches with the aim of filling the knowledge gap on GC in these areas.

**Methods:**

This observational study has three parts that are conducted in parallel during 2019–2023 across recruiting centers from four EU and four LATAM countries: Part 1) A case-control study (800 cases and 800 controls) using questionnaires on candidate risk factors for GC, which will be correlated with clinical, demographic and epidemiological parameters. Part 2) A case-control tissue sampling study (400 cases and 400 controls) using proteome, genome, microbiome and immune analyses to characterize advanced (stage III and IV) GC. Patients in this part of the study will be followed over time to observe clinical outcomes. The first half of samples will be used as training cohort to identify the most relevant risk factors and biomarkers, which will be selected to propose cost-effective diagnostic and predictive methods that will be validated with the second half of samples. Part 3) An educational study, as part of our prevention strategy (subjects recruited from the general public) to test and disseminate knowledge on GC risk factors and symptoms by a questionnaire and informative video. Patients could be recruited for more than one of the three LEGACy studies.

**Discussion:**

The LEGACy study aims to generate novel, in-depth knowledge on the tumor biological characteristics through integrating epidemiological, multi-omics and clinical data from GC patients at an EU-LATAM partnership. During the study, cost-effective panels with potential use in clinical decision making will be developed and validated.

**Trial registration:**

ClinicalTrials.gov Identifiers:

Part 1: NCT03957031.

Part 2: NCT04015466.

Part 3: NCT04019808.

**Supplementary Information:**

The online version contains supplementary material available at 10.1186/s12885-022-09689-9.

## Background

Gastric Cancer (GC) is the fifth most common and fourth most deadly cancer worldwide [1]. Risk factors associated with the development of GC include *H. pylori (HP)* infection, unhealthy lifestyle habits including obesity, smoking, consumption of alcohol and processed meat. Other risk factors associated with the development of GC include atrophic gastritis, partial gastrectomy, and inherited genetic predisposition [2]. Patients with GC report a variety of symptoms including indigestion, abdominal pain, changes in bowel movement habits, weight loss and fatigue. However, symptoms are often hard to interpret and in over half of cases tumors have already metastasized to regional lymph nodes or distant locations by the time they are recognized. At this advanced stage of the disease, systemic treatment is only limited effective and associated with a median overall survival of 11 months [3, 4].

Furthermore, geographic variation in the incidence of GC have been reported. The global burden of gastric cancer is ﻿over 1,000,000 new cases and 769,000 deaths per year. Gastric cancer age standardized incidence rates are highest in Eastern Asia with rates of 32.5 for males and 13.2 for females. Most extensive studies have been performed on Northern American and Asian populations, which have led to better prevention and screening strategies. For example in Japan 2/3 of the incidence rates are early stage cancers, leading to declining dead rates [5]. Also in other parts of the world such as South America and Eastern and Southern Europe gastric cancer is a prevalent disease. However, these populations are often underrepresented in molecular profiling studies and clinical trials and therefore not completely understood [1]. Geographic variations in incidence rates are seen between but also within continents [6]. This could be illustrated by the differences in mortality in Chile and its neighboring country Argentina: The number one cancer death cause for Chilean males is GC, while for males in Argentina, GC is ranked in the fifth place. For females from these countries an opposite trend is observed. Several studies have suggested there may be a strong environmental component explaining these regional variations in GC incidence and mortality, as well as in age, obesity and lifestyle [6–8]. However, direct comparisons in risk factors between countries is challenging as most of this knowledge comes from patient data included in different studies [9, 10]. Great geographical differences may also be explained by differences that are observed in prevalence and resistance rates of HP, which infects 50% of the world population and is the most important risk factor of GC [11, 12]. Furthermore, epidemiological and molecular features of GC vary widely by histological type, location, and genetic makeup of the tumor among patients worldwide [13].

There have been multiple attempts to increase our understanding of the molecular drivers of GC [14, 15]. Studies from The Cancer Genome Atlas (TCGA) project and The International Cancer Genome Consortium (ICGC), for instance, have identified four molecular subgroups based on genomic, epigenetic, transcriptomic and proteomic data: tumors positive for Epstein-Barr virus, microsatellite instable tumors, genome stable cancers and tumors with chromosomal instability [14, 16]. These subgroups differ in molecular and immunological features [17], which are associated with prognosis and response to treatment [18–20]. TCGA profiling has mostly been via analysis of non-advanced gastrectomy samples (stages I-II). Much less is known about the molecular profile of advanced cancers (stage III-IV) and cancers located at the gastroesophageal junction (GEJ). Furthermore, as patients from European (EU) and Latin American (LATAM) countries are underrepresented in these studies, it is unknown whether the molecular subtypes are fully representative of these populations [21]. Finally, available multi-omic classification procedures are too difficult and expensive to be implemented in the clinical setting, especially in LATAM countries.

All together these studies showed that a better understanding of the key oncogenic drivers and epidemiological factors are needed in GC from European and LATAM countries to improve GC prevention therapeutic approaches. To accomplish this goal we have set up the LEGACy study: a consortium between 11 research institutes from European and Latin American countries with the goal to 1) identify and comparing GC characteristics and associated risk factors between GC patients and healthy controls in EU and LATAM populations due to identify high-risk groups with a view to tailoring early detection and diagnosis; 2) increase our understanding of advanced GC tumor biology in EU-LATAM from a multi-omics approach that will provide extensive information including the tumor’s pathologic, genetic, immunologic and microbiome characteristics. This could lead to clinically relevant diagnostic or prognostic biomarkers and potential targets for therapy; 3) prevention by measuring and educate the general population on GC risk factors and symptoms and promoting healthy lifestyle habits with the ultimate goals to improve GC outcomes. Understanding the regional variations in biological and clinical behavior of GC will help to create a foundation for globally implementable diagnostic and treatment approaches.

## Methods/design

### Patients

Patients with GC and GEJ cancer will be recruited from eight university medical centers in seven different countries in EU (Spain, Netherlands, and Portugal) and LATAM (Argentina, Mexico, Chile, and Paraguay) from 2019 to 2023. Advanced stages will include state III and IV according to the American Joint Committee on Cancer (AJCC) stage system, GEJ will include Siewert type I and II tumors [22]. The clinical sites were selected based on their interest, expertise and geographical location, as well as the recruitment potential for the purpose of this study. Patient recruitment for each study is performed by coordinating a multidisciplinary team in each center including gastroenterologists, pathologists, surgeons, and medical oncologists. All patients should go through the informed consent procedure approved by each Institutional Research Board to participate in each of the sub studies.

### Institutions and partners

**Fig. 1:**
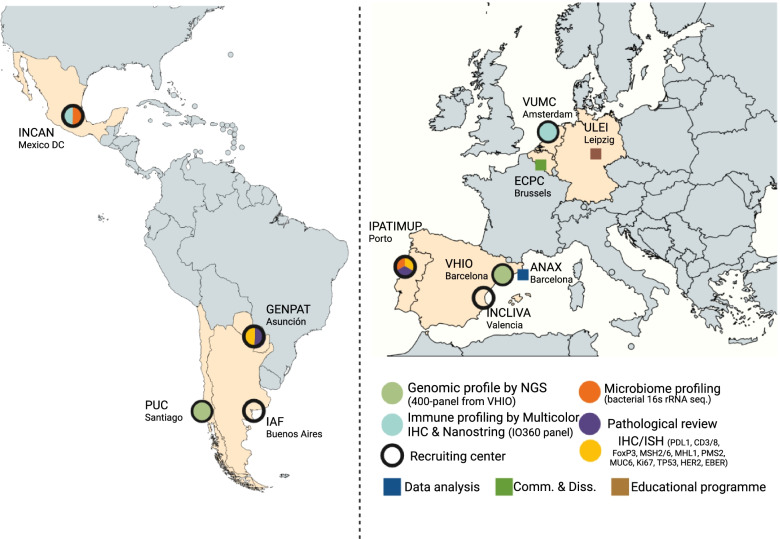
Overview LEGACy; Indicating which centers contribute to which determination and related parts of the project. ANAX: Anaxomics SL (Spain); ECPC: European Cancer Patient Coalition (Belgium); GENPAT: Genpat SL (Paraguay); IAF: Institute Alexandre Fleming (Argentine); INCAN: Caner Insitute of Mexico (Mexico); INCLIVA: Biomedical Research Center INCLIVA (Spain); PUC: Pontificia University of Chile (Chile); ULEI: University of Leipzig (Germany); VHIO: Valld’Hebrón Insitut of Oncology (Spain); VUMC: Amsterdam UMC (Netherlands). IHC: Immunohistochemistry; ISH: In situ hybridization. Map created with mapchart.net. Figure created with BioRender.com

LEGACy is a multi-institutional research approach performed by a team of four LATAM and seven EU organizations. The study members and centers can be found in Fig. 1 and in Supplementary Table 1. To ensure homogeneity and reproducibility of data collection by all different project partners the recruitment of subjects and the collection and handling of patient material and data has been standardized via use of a laboratory handbook. This handbook provides standard operating procedures on all processes and procedures pertaining to subjects, samples and assays included in the LEGACy project. All researchers, doctors, nurses, and data managers receive training on correct questionnaire administration, clinical data collection and biopsy collection before they begin work in the project.

### Study design

#### LEGACy part 1

**Fig. 2:**
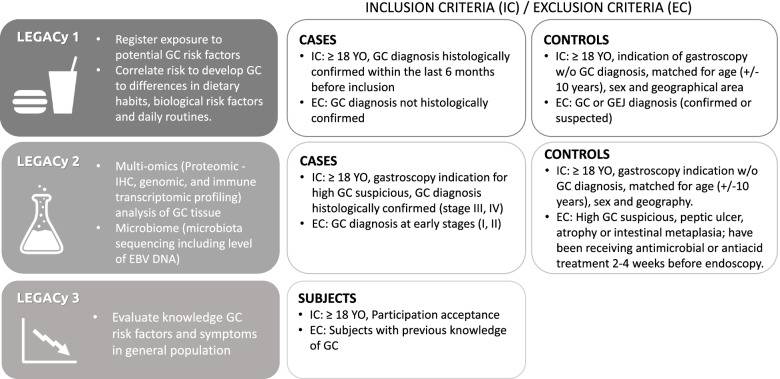
Subjects, inclusion criteria (IC) and exclusion criteria (EC) for three sub-studies of the LEGACy project. (YO: years old; ICS: Informed consent sheet; GC: gastric cancer; GEJ: gastro-esophageal junction cancer; w/o: without)

**LEGACy part 1** is a case control study focused on identifying geographic variations in known and potential risk factors including detailed food consumption habits, BMI, socioeconomic status, smoking and alcohol use and family history of cancer. Within the study, cases are defined as adults with histologically confirmation of stage I-IV gastric adenocarcinoma (including GEJ cancer) within 6 months prior to inclusion, while controls are patients without gastric cancer from the same geographic regions as the cases, undergoing gastroscopy to rule out malignant disease. After signing informed consent forms, participants will be called by a trained member of the local LEGACy team who will record responses to the 30 min questionnaire in the eCRF. Epidemiologic data will be collected through a questionnaire. This questionnaire was modified and adapted according to the populations epidemiological facts of these regions to a shorter version to make a feasible approach in a 30 min call, including the most representative variables to measure and compare risk factors associated in EU and LATAM populations. A list of the parameters is provided in supplement 2. The data on risk factors will be compared between the different geographic regions and correlated with corresponding clinical data including age, sex, histology, disease stage and outcomes. Each recruiting center will include 100 cases and 100 control patients for the questionnaire, resulting in a total recruitment of 800 cases and 800 controls. Patients of this study are allowed to participate in other LEGACy studies when meeting all inclusion criteria as shown in Fig. 2.

#### LEGACy part 2

**LEGACy part 2** is a tumor tissue sampling study focusing on multi-omics characterization of advanced GC in EU and LATAM populations. Data analyses include immunohistochemistry (IPATIMUP and GENPAT), genomics (VHIO and PUC), the immunome (VUMC and INCAN) and microbiome (IPATIMUP and PUC) of which the details can be found in Fig. 1. The patient population targeted of this study are treatment-naive advanced (stage III-IV) GC or GEJ adenocarcinomas. Subjects with gastroscopy indicated for a benign disease and confirmed absent of GC will be invited to participate as study controls.

From each patient included as a case eight tumor biopsies will be collected in formalin during gastroscopy, and in case of surgical tumor removal the resection specimen can be used as substitute. In cases, additional biopsies will be collected from non-tumoral gastric mucosa areas within four cm (2 in formalin, 1 snap frozen) and more than four cm around the tumor (2 in formalin). For controls, biopsies will be collected from gastric mucosa tissue at the corpus (2), antrum (2), and incisura (1) in formalin, and one extra antrum biopsy will be snap frozen in liquid nitrogen. For microbiome analysis, one of the gastric mucosa biopsies from each patient will be snap frozen in liquid nitrogen. Blood samples will be collected in one serum and 2 EDTA tubes of 6-10 ml each at the time of gastroscopy to isolate plasma, serum and non-viable peripheral blood mononuclear cells from both cases as controls. The biopsies collected and fixed in formalin will be paraffin-embedded (FFPE). All sample collections will be done in a standardized matter following a uniform lab manual. All FFPE tissues will be centrally collected at one destinated center, subjected to pathological examination by an expert pathologist and then distributed for further analyses. QC on each sample before and after RNA and DNA extraction will be performed, according to the different protocol requirements by the expert centers who perform the multi-omic determinations as indicated in Fig. 1.

Our first aim is to identify potential molecular subgroups that can be attributed to existing clinically relevant classification models, such as the TCGA molecular subgroups. Patients will therefore be stratified into subgroups based on the multi-omic variables that have tumor-driving potential and are distinctive for each specific subgroup. These features will be correlated with their corresponding clinical, pathological, and epidemiological characteristics. As a multi-omics method is not economically or practically applicable in clinical setting, a final cost-effective set of the most clinically relevant biomarkers will be proposed for validation in clinical practice.

The first half of the patients will be considered as training cohort. After analyzing the results of this cohort, the most relevant molecular biomarkers will be converted into more cost-effective panels and validated in the second half of the patients: the validation cohort. Based on GC incidence and presentation of advanced GC at each participating center in this proposal for the duration of 3 to 4 years it was decided to include 50 cases and 50 controls of each center (total 400 cases and 400 controls).

### LEGACy part 3

This study consists of an online module for recording the knowledge of the general population on GC risk factors and symptoms and to provide education, as certain populations are reported to have low knowledge on risk factors [23]. The strategy is organized as follows: a) The participants consent their participation through an online informed consent form. b) The participants complete an online questionnaire on their knowledge of gastric cancer risk factors and symptoms and this information is recorded for further analysis by the LEGACy group. c) After completing the questionnaire, the subjects receive an informational brochure and a short video containing essential information about GC. d) After that, the same online questionnaire is provided after a few months to record the short- and long-term impact of this educational approach. The questionnaire lasts 15 minutes and was prepared by the LEGACy consortium partners and validated by the European Cancer Patient Coalition organization. The questionnaire and video is available in English, Spanish, Portuguese and Dutch. Finally, throughout this study, knowledge and best practices will be shared though educational training programs to share members’ expertise and train all involved project members. The courses will cover different aspects of GC including epidemiology, pathology, diagnosis, current and future treatments. Moreover, open access links to the online training courses are disseminated through the LEGACy website and social media channels to reach anyone interested to learn more about gastric cancer, to make a continuous and durable impact in GC research and clinical management.

### Sample size considerations

Since there are a large number of specific factors to be analyzed with multiple techniques, and this is mostly a descriptive study, formal sample size calculations for the study as a whole were not feasible. The number of patients to include in the studies was decided upon from the following considerations:

(i) LEGACy part 1, 2 and 3 study sample size was based on the participating clinical centers capacity of recruitment along the project.
(ii) To get a better idea what sample size would be needed to give relevant results, we made an example calculation based on a RNA experiment in the two main groups. For example, we are planning to identify differential gene expression between two groups. Prior data indicates that the minimum average read counts among the differential genes in the control group is 5, the maximum dispersion is 0.5, and the ratio of the geometric mean of normalization factors is 1. Suppose that the total number of genes for testing is 20,000 and the top 100 genes are differentially expressed. If the desired minimum fold change is 1.5, we will need to study 200 subjects in each group to be able to reject the null hypothesis that the population means of the two groups are equal with probability (power) 0.8 using exact test. The FDR associated with this test of this null hypothesis is 0.01.
(iii) Based on (i) and the calculation in (ii) it was decided to include 200 cases and 200 controls for the training cohort, and to add another 200 cases and 200 controls for the validation cohort in LEGACy part 2 to be sure the generated results would be usable.
(iv) LEGACy part 1 is not limited to GC stage of disease. Therefore, it was decided to include two times the amount of patients included in part 2.

## Discussion

The LEGACy study is an extensive epidemiologic and translational study in GC that is conducted in an EU-LATAM partnership. Our study will provide novel, detailed and comparable data on epidemiological and biological features of GC. The LEGACy project will generate comprehensive data that could complement the limited information on advanced GC, the biological role of anatomic location of the tumor and balance the underrepresentation of GC patients from EU and LATAM countries in large molecular studies. Furthermore, this study also provides knowledge on risk factors that could help to enhance prevention of gastric cancer. The limitations of our study are: 1) data are obtained at multiple sites and therefore SOPs need to be followed precisely; 2) our study includes four EU countries and four LATAM countries, so caution should be taken in extrapolating data to other EU and LATAM areas not included in the study. Even though multiple handbooks, standard operation procedures, protocols and trainings are in place to guarantee correct subject/sample processing and to assure comparable quality of samples, minimizing data heterogeneity will be an ongoing focus of attention. Despite the extensive character of this study, the techniques used in the training cohort have shown to be feasible as have resulted in good quality data of all multi-omics techniques of almost all samples that have been processed so far. With this ambitious project we attempt to provide better understanding of clinicopathological details, molecular features and risk factors and develop important collaborative infrastructures which is crucial in our global attempt to provide precision medicine to patients with GC.

## Supplementary Information

**Additional file 1.**

**Additional file 2.**

## Data Availability

The datasets that will be generated and/or analyzed during the current study are available from the corresponding author upon reasonable request.

## Abbreviations

GC: Gastric Cancer
EU: European
LATAM: Latin American
HP: *H. pylori*
TCGA: The Cancer Genome Atlas
GEJ: Gastroesophageal Junction
FFPE: formalin-fixed paraffin-embedded
ANAX: Anaxomics SL (Spain)
ECPC: European Cancer Patient Coalition (Belgium)
GENPAT: Genpat SL (Paraguay)
IAF: Institute Alexandre Fleming (Argentine)
INCAN: Cancer Insitute of Mexico (Mexico)
INCLIVA: Biomedical Research Center INCLIVA (Spain)
IPATIMUP: Institute of Molecular Pathology and Immunology of the University of Porto (Portugal)
PUC: Pontificia University of Chile (Chile)
ULEI: University of Leipzig (Germany)
VHIO: Vall d’Hebrón Institute of Oncology (Spain)
VUMC: Amsterdam University Medical Center (Netherlands)
