# Supplementary material for: The LEGACy study: a European and Latin American consortium to identify risk factors and molecular phenotypes in gastric cancer to improve prevention strategies and personalized clinical decision making globally
Source: BMC Cancer. 2022 Jun 13;22:646. doi: 10.1186/s12885-022-09689-9 (PMC9190072; doi:10.1186/s12885-022-09689-9)
Supplement: Supplementary file 1 — Additional file 1. [file 12885_2022_9689_MOESM1_ESM.docx]

**Annex table 1: Study sites and study members**

| Amsterdam UMC, Department of Medical Oncology, Cancer Center Amsterdam, Amsterdam, The Netherlands and Oncode Institute | De boelelaan, 1117  1081 HV Amsterdam, The Netherlands | Tessa S. van Schooten |
| --- | --- | --- |
|  |  | Sarah Derks |
|  |  | Roos E. Pouw |
| Instituto Investigación Sanitaria INCLIVA (INCLIVA), Medical Oncology Department, Hospital Clínico Universitario de Valencia | Avenida Blasco Ibáñez, 17, 46010, Valencia, Spain | Tania Fleitas Kanonnikoff |
|  |  | Andrés Cervantes Ruipérez |
|  |  | Elena Jiménez-Martí |
|  |  | Valentina Gambardella |
|  |  | Dolores Iglesias Ferri |
|  |  | Juan A. Carbonell Asins |
|  |  | Andrés Peña |
|  |  | Pablo Navarro |
|  |  | Rosana Viaplana |
|  |  | Manuel Cabeza Segura |
|  |  | Beatriz López |
| Institute of Pathology and Immunology of University of Porto. Department of Anatomic Pathology, Centro Hospitalar São João | Alameda Prof. Hernâni Monteiro  4200-319 Porto, Portugal | Fatima Carneiro |
|  |  | Ceu Figueiredo |
|  |  | Rita Barros |
|  |  | Rui Ferreira |
| Instituto Nacional de Cancerología (INCAN), Translational Medicine Laboratory & GI Cancer Department | San Fernando N.22, Colonia Seccion XVI,  14080, Mexico City, Mexico | Erika Ruiz |
|  |  | Edith Fernández Figueroa |
|  |  | Alberto Leon-Takahashi |
|  |  | Saul Lino-Silva |
|  |  | Angelica Hernandez-Guerrero |
|  |  | Consuelo Diaz-Romero |
|  |  | Nayelli Ortiz-Olvera |
|  |  | Claudia Rangel Escareño |
|  |  | Melissa Valdez-Reyez |
|  |  | Uriel Coquis-Navarrete |
| Valld’Hebron Institute of Oncology (VHIO), Medical Oncology Department | Valld’Hebron University Hospital  Centro Cellex, CalleNatzaret, 115-117  08035 Barcelona, Spain | Maria Alsina |
|  |  | Cristina Molero |
|  |  | Rodrigo Dienstmann |
|  |  | Josep Maria Miquel |
| Faculty of Dentistry, Universidad de los Andes, Santiago, Chile | Diagonal Paraguay 362  8330077, Santiago, Chile | Ignacio Retamal |
| Pontificia Universidad Católica de Chile, Departamento de Hematología Oncología, Facultad de Medicina | Diagonal Paraguay 362  8330077, Santiago, Chile | Marcelo Garrido |
|  |  | Arnoldo Riquelme |
|  |  | Mauricio Pinto |
|  |  | Matias Muñoz |
|  |  | Maria Loreto Bravo |
|  |  | Miguel Cordova |
|  |  | Valentina Ortiz |
|  |  | Margarita Pizarro |
|  |  | Gareth Owen |
| GenPat | Guido Spano 1448 e/ Ohiggins y Dr. Morra, Asunción, Paraguay | Carmelo Caballero |
|  |  | Hugo Boggino |
| Instituto de Previsión Social | Constitución esq. Luis A. de Herrera, Asunción, Paraguay | Eva Lezcano |
|  |  | Daniel Cantero |
|  |  | Cinthia Gauna |
|  |  | Horacio Lezcano |
|  |  | Maria Rita Pereira |
|  |  | Mariela Romero |
|  |  | Loida Caballero |
| Instituto Alexander Fleming,  Medical Oncology Department | Avenida Crámer, 1180C1426ANZ Buenos Aires, Argentina | Federico Esteso |
|  |  | Juan Manuel O’Connor |
|  |  | Romina Luca |
| Anaxomics Biotec | Anaxomics Biotech, S.L. C/ Diputació 237, 1-1, 08007 Barcelona, Spain | Judith Farrés |
|  |  | José Manuel Mas |
| Medsci Institute | Cruz Verde 44 interior 9 col. San Nicolás Totolapan, Magdalena Contreras, Ciudad de México, CP 10900 | Juan Carlos Falcon |
| University Cancer Center Leipzig (UCCL), Leipzig University Medical Center,.Department of Oncology, Gastroenterology, Hepatology, Pulmonology, and Infectious Diseases | Liebigstraße 22, 04103 Leipzig, Germany | Florian Lordick |
|  |  | Jeannette Vogt |
| European Cancer Patient Coalition, Direction& EU Affairs Dpt. | Avenue des Arts 6, B-1210, Brussels, Belgium | Antonella Cardone |
|  |  | Charis Girvalaki |
