# Supplementary material for: The LEGACy study: a European and Latin American consortium to identify risk factors and molecular phenotypes in gastric cancer to improve prevention strategies and personalized clinical decision making globally
Source: BMC Cancer. 2022 Jun 13;22:646. doi: 10.1186/s12885-022-09689-9 (PMC9190072; doi:10.1186/s12885-022-09689-9)
Supplement: Supplementary file 2 — Additional file 2. [file 12885_2022_9689_MOESM2_ESM.docx]

**Subject ID:__________________________________________________________**

General Information (Questionnaire 1)

NOTE: Any numerical question which answer is unknow or never, introduce the code 98

**I. Basic information**

| **Question#** | **Data Element Label** | **Data Entry Alternatives** | | | |
| --- | --- | --- | --- | --- | --- |
| 1 | Gender |  | | Male | |
|  |  |  | | Female | |
| 2 | Year of birth | Year | | | ______ (YYYY) |
| 3 | What is your race/ethinicity? |  | American Indian | | |
|  |  |  | Asian | | |
|  |  |  | White Hispanic or Latino | | |
|  |  |  | White Not-Hispanic or Latino | | |
|  |  |  | Black or African American | | |
|  |  |  | Not reported | | |
|  |  |  | Unknown | | |

**II. Anthropometrics and performance status**

| **Question#** | **Data Element Label** | **Data Entry Alternatives** | |
| --- | --- | --- | --- |
| 1 | How tall are you? |  | __________ cm |
| 2 | How much do you weight? |  | __________ Kg |
| 3 | On a scale of 0 to 4, how would you rate your overall health?  (Being 0 excellent and 4 very Bad) | 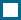 | 0 Excellent |
|  |  | 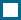 | 1 Good |
|  |  | 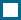 | 2 Moderate |
|  |  | 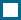 | 3 Bad |
|  |  | 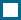 | 4 Very Bad |
|  |  | 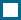 | 5 Prefer not to answer |

**III. General Clinical Data**

| **Question#** | **Data Element Label** | **Data Entry Alternatives** | |
| --- | --- | --- | --- |
| 1 | Does the patient have any gastric symptoms? | 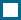  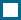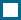 | Yes  No  Unknown |
| 2 | Is the patient receiving any antibiotic treatment in the last 30 days? | 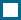  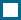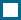 | Yes  No  Unknown |
| 3 | Did the patient receive any anti-reflux treatment in the last 30 days? | 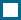  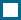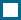 | Yes  No  Unknown |

**V. Other malignancy form**

| **Question#** | **Data Element Label** | **Data Entry Alternatives** | | | |
| --- | --- | --- | --- | --- | --- |
| 1 | Have you had another form of previous cancer? | 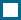 | YES, if so, please answer questions 2,3 and 4 | | |
|  |  | 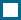 | NO | | |
|  |  | 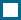 | Prefer not to answer | | |
| 2 | Date of Diagnosis | - 1. Day | | | _______ (DD) |
|  |  | - 1. Month | | | _______ (MM) |
|  |  | - 1. Year | | | _______ (YYYY) |
| 3 | Primary site of disease (NOTE: the site of any prior malignancy cannot be the same site if the primary tumor submitted to LEGACy) | _______________________ (Specify) | | | |
| 4 | Did patient undergo curative treatment for this malignancy? | 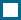 | | Yes | |
|  |  | 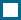 | | No | |

Epidemiology Information (Questionnaire 2)

**V. Background, demographics, household feature and family history of gastric cancer**

| 1 | What is the highest level of school that you have completed? | 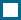 | No more than primary (grades 1-8) | | | |
| --- | --- | --- | --- | --- | --- | --- |
|  |  | 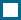 | Some high school (grades 9-11) | | | |
|  |  | 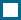 | High school graduate or GED | | | |
|  |  | 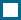 | Technical or trade school | | | |
|  |  | 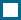 | Some college | | | |
|  |  | 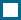 | College graduate | | | |
|  |  | 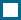 | Graduate school | | | |
|  |  | 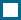 | Other(Specify): _________________ | | | |
|  |  | 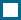 | Prefer not to answer | | | |
| 2 | What is the location of your residence? | 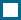 | Urban | | | |
|  |  | 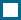 | Rural | | | |
|  |  | 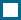 | Prefer not to answer | | | |
| 3 | How many years have you lived in your current residence? |  | | _________ years | | |
|  |  | 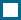 | | Prefer not to answer | | |
| 4 | What is your primary source of drinking water at your current residence? | 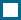 | Tap water from a public/municipal water utility | | | |
|  |  | 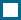 | Private well | | | |
|  |  | 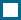 | Other (Specify): ____________________ | | | |
|  |  | 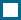 | Prefer not to answer | | | |
| Do you have any of the following items in your home? | | | | | | |
|  | Item | Yes | | | No | Prefer not to answer |
|  | Automobile | 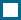 | | | 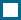 | 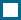 |
| 5 | Cellphone | 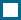 | | | 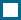 | 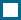 |
|  | Laptop/PC | 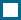 | | | 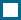 | 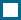 |
|  | Internet | 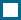 | | | 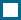 | 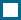 |
|  | Television with cable | 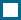 | | | 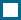 | 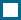 |
|  | Refrigerator | 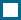 | | | 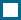 | 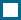 |
|  | Microwave | 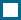 | | | 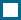 | 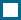 |
| 6 | What is the main fuel used for cooking in your residence? | 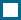 | Gas | | | |
|  |  | 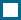 | Electricity | | | |
|  |  | 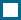 | Charcoal | | | |
|  |  | 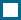 | Firewood | | | |
|  |  | 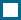 | Other (Specify): ____________________ | | | |
|  |  | 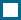 | Prefer not to answer | | | |
| 7 | What type of central heating system is available in your residence? | 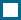 | Gas | | | |
|  |  | 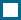 | Electricity | | | |
|  |  | 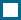 | Kerosene | | | |
|  |  | 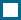 | Firewood | | | |
|  |  | 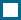 | Other (Specify):____________________ | | | |
|  |  | 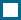 | No heating available | | | |
|  |  | 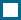 | Prefer not to answer | | | |
| 8 | What is your current working status? | 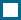 | Employed | | | |
|  |  | 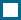 | Unemployed | | | |
|  |  | 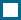 | Household | | | |
|  |  | 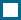 | Retired | | | |
|  |  | 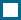 | Other (Specify): ___________________ | | | |
|  |  | 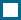 | Prefer not to answer | | | |
| 9 | How many people currently live in your household, please include yourself in the total number? |  | __________ | | | |
|  |  | 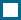 | Prefer not to answer | | | |
| 10 | How many siblings do you have, including full and half-siblings? [If you are not sure, enter 98] |  | __________ | | | |
|  |  | 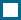 | Prefer not to answer | | | |
| 11 | Do you have any first (i.e., parents, sibling, children) or second degree (i.e., uncles, aunts, nephews, nieces, grandparents, grandchildren, and half-siblings) relatives who were diagnosed with stomach cancer? | 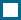 | No | | | |
|  |  | 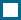 | Yes. Specify, who? | | | |
|  |  | 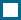 | Prefer not to answer | | | |
| 12 | At which age were your relatives diagnosed with stomach cancer? (if more than one case of stomach cancer, please specify the youngest age) |  | __________ | | | |
|  |  | 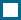 | Prefer not to answer | | | |

**VI. Cigarette consumption**

| **Question#** | **Data Element Label** | **Data Entry Alternatives** | | |
| --- | --- | --- | --- | --- |
| 1 | Have you ever smoked? | 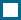 | No | |
|  |  | 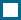 | Yes | |
| 2 | Have you smoked at least 100 cigarettes (5 packs) in your entire life? | 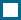 | | No |
|  |  | 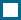 | | Yes |
| 3 | How old were you when you first smoked a cigarette? |  | | __________ years |
| 4 | How often do you smoke cigarettes? | 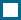 | | Every day |
|  |  | 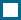 | | Some days. Please specify the number of days per week: ________ |
|  |  | 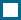 | | Not at all. How old were you when you last smoked? ________ years |

**VII. Alcohol consumption**

| **Question#** | **Data Element Label** | **Data Entry Alternatives** |
| --- | --- | --- |

| 1 | Have you ever consumed regularly (at least one drink per month for one year)? | 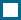 | YES, if so, please answer questions 2 to 7 |
| --- | --- | --- | --- |
|  |  | 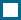 | NO |
| 2 | How old were you when you first drank alcohol regularly (at least one drink per month for one year)? |  | ____________years |

| 3 | Which one of the following best describes your current alcohol drinking pattern? | 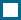 | Current drinker, I only drink during weekdays | |
| --- | --- | --- | --- | --- |
|  |  | 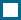 | Current drinker, I only drink during weekends | |
|  |  | 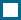 | Current drinker, I drink everyday | |
|  |  | 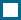 | Former drinker (then answer 3.1 and 3.2, 4 and 5) | |
| 3.1 | At what age did you last drink alcohol |  | ________ years | |
| 3.2 | Why did you stop drinking alcohol (Mark all that apply)? | 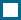 | Personal choice | |
|  |  | 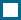 | Health or medical reasons | |
|  |  | 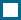 | Interactions with medications | |
|  |  | 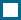 | Other (Specify): | ________________________ |
| 4 | Which is/was your mean weekly alcohol consumption? (Select number (1-3) that best describes your mean weekly consumption) |  | 1: 1 to 5 drinks per week | |
|  |  |  | 2: 5 to 10 drinks per week | |
|  |  |  | 3: more than 10 drinks per week | |
| 5 | Which type of alcoholic drinks dou you drink frequently/on a day? (mark all that apply) |  | Wine | |
|  |  |  | Beer | |
|  |  |  | Distilled (spirits) | |

**VIII. Nutritional and lifestyle habits**

| **During the past six – twelve months, did you eat any of the following?**  **IF YES, ASK HOW OFTEN. If the periodicity is other than the specified, then mark Occasionally.**  **IF NO, CIRCLE ZERO** | | | | | | |  |
| --- | --- | --- | --- | --- | --- | --- | --- |
|  | Zero | 1-2 times per **week** | 3 + times per **week** | 1 time a **day** | 2 times a **day** | 3+times a **day** | Occasi-onally |
| White bread / white bread rolls | 0 | 1 | 2 | 3 | 4 | 5 | 6 |
| Brown /wholewheat bread / Rolls | 0 | 1 | 2 | 3 | 4 | 5 | 6 |
| Breakfast Cereal (processed) | 0 | 1 | 2 | 3 | 4 | 5 | 6 |
| Breakfast Cereal (minimally processed - weetbix, muesli, etc.) | 0 | 1 | 2 | 3 | 4 | 5 | 6 |
| Crackers (ProVita etc) | 0 | 1 | 2 | 3 | 4 | 5 | 6 |
| Cookies, biscuits,rusks | 0 | 1 | 2 | 3 | 4 | 5 | 6 |
| Cake / scone / muffin / puddings / pancake / fruit pie / koeksister | 0 | 1 | 2 | 3 | 4 | 5 | 6 |
| Roti / samoosa / springroll / doughnut | 0 | 1 | 2 | 3 | 4 | 5 | 6 |
| Pizza | 0 | 1 | 2 | 3 | 4 | 5 | 6 |
| Pasta/noodle dishes with cheese sauces (macaroni cheese, lasagne, noodle salad etc.) | 0 | 1 | 2 | 3 | 4 | 5 | 6 |
| Popcorn | 0 | 1 | 2 | 3 | 4 | 5 | 6 |
| Crisps (Simba and Niknaks etc.) | 0 | 1 | 2 | 3 | 4 | 5 | 6 |
| Beef sausage (boerewors) | 0 | 1 | 2 | 3 | 4 | 5 | 6 |
| Polony /salami / bacon / salami / pork sausages (processed meat, cooked, smoked and canned) | 0 | 1 | 2 | 3 | 4 | 5 | 6 |
| Meat or chicken pies/sausage rolls | 0 | 1 | 2 | 3 | 4 | 5 | 6 |
| Chicken - battered (KFC etc). and chicken burger only | 0 | 1 | 2 | 3 | 4 | 5 | 6 |
| Meat and meat dishes (steaks, minced meat, cottage pie, mince, meatballs, stew, bobotie, etc.) | 0 | 1 | 2 | 3 | 4 | 5 | 6 |
| Gravy, made with stock or gravy powder | 0 | 1 | 2 | 3 | 4 | 5 | 6 |
| Biltong/dry wors /fish biltong | 0 | 1 | 2 | 3 | 4 | 5 | 6 |
| Milk (all types, also dairy fruit juice, malted milk, milk shakes) | 0 | 1 | 2 | 3 | 4 | 5 | 6 |
| Maas (fermented milk) | 0 | 1 | 2 | 3 | 4 | 5 | 6 |
| Cheese | 0 | 1 | 2 | 3 | 4 | 5 | 6 |
| Yoghurt | 0 | 1 | 2 | 3 | 4 | 5 | 6 |
| Eggs | 0 | 1 | 2 | 3 | 4 | 5 | 6 |
| Tinned fish (pilchards/tuna, etc.) | 0 | 1 | 2 | 3 | 4 | 5 | 6 |
| Other fish and seafood | 0 | 1 | 2 | 3 | 4 | 5 | 6 |
| Potato chips/french fries and potato salad | 0 | 1 | 2 | 3 | 4 | 5 | 6 |
| Canned vegetables, incl. Baked beans, tomato paste, sweetcorn, etc | 0 | 1 | 2 | 3 | 4 | 5 | 6 |
| Soup (all types) | 0 | 1 | 2 | 3 | 4 | 5 | 6 |
| Salad dressing/mayonnaise | 0 | 1 | 2 | 3 | 4 | 5 | 6 |
| Ice cream (all types) | 0 | 1 | 2 | 3 | 4 | 5 | 6 |
| Margarines, all types, also butter | 0 | 1 | 2 | 3 | 4 | 5 | 6 |
| Chutney / atchar / chakalaka / Worcester sauce | 0 | 1 | 2 | 3 | 4 | 5 | 6 |
| Savoury sauces (mushroom, monkey gland, white, cheese) | 0 | 1 | 2 | 3 | 4 | 5 | 6 |
| Tomato sauce | 0 | 1 | 2 | 3 | 4 | 5 | 6 |
| Salt | 0 | 1 | 2 | 3 | 4 | 5 | 6 |
| Aromat / Fondor /mustard | 0 | 1 | 2 | 3 | 4 | 5 | 6 |
| Peanuts | 0 | 1 | 2 | 3 | 4 | 5 | 6 |
| Peanut butter | 0 | 1 | 2 | 3 | 4 | 5 | 6 |
| Marmite/Bovril | 0 | 1 | 2 | 3 | 4 | 5 | 6 |
| Chocolate sweets and sauce | 0 | 1 | 2 | 3 | 4 | 5 | 6 |
| Coffee | 0 | 1 | 2 | 3 | 4 | 5 | 6 |
| Vinegar | 0 | 1 | 2 | 3 | 4 | 5 | 6 |
| Hot beberages | 0 | 1 | 2 | 3 | 4 | 5 | 6 |

| **Do you even eat? (Yes=1; No=0)** | | | |
| --- | --- | --- | --- |
| 1 | CEREALS | corn/maize, rice, wheat, sorghum, millet or any other grains or foods made from these (e.g. bread, noodles, porridge or other grain products) + insert local foods e.g. ugali, nshima, porridge or pastes or other locally available grains |  |
| 2 | VITAMIN A RICH VEGETABLES AND  TUBERS | pumpkin, carrots, squash, or sweet potatoes that are orange inside + other locally available vitamin-A rich vegetables (e.g. red sweet pepper) |  |
| 3 | WHITE TUBERS AND ROOTS | white potatoes, white yams, white cassava, or other foods made from roots |  |
| 4 | DARK GREEN LEAFY VEGETABLES | dark green/leafy vegetables, including wild ones + locally available vitamin-A rich leaves such as amaranth, cassava leaves, kale, spinach etc. |  |
| 5 | OTHER VEGETABLES | other vegetables (e.g. tomato, onion, eggplant) , including wild vegetables |  |
| 6 | VITAMIN A RICH FRUITS | ripe mangoes, cantaloupe, apricots (fresh or dried), ripe papaya, dried peaches + other locally available vitamin A-rich fruits |  |
| 7 | OTHER FRUITS | other fruits, including wild fruits |  |
| 8 | ORGAN MEAT (IRON-  RICH) | liver, kidney, heart or other organ meats or blood-based foods |  |
| 9 | FLESH MEATS | beef, pork, lamb, goat, rabbit, wild game, chicken, duck, or other birds |  |
| 10 | EGGS | chicken, duck, guinea hen or any other egg |  |
| 11 | FISH | fresh or dried fish or shellfish |  |
| 12 | LEGUMES, NUTS AND SEEDS | beans, peas, lentils, nuts, seeds or foods made from these |  |
| 13 | MILK AND MILK  PRODUCTS | milk, cheese, yogurt or other milk products (from animal origin) |  |
| 14 | OILS AND FATS | oil, fats or butter added to food or used for cooking |  |
| 15 | RED PALM PRODUCTS | Red palm oil, palm nut or palm nut pulp sauce. Including industrial baking like donuts and processed cereals |  |
| 16 | SWEETS | sugar, honey, sweetened soda or sugary foods such as chocolates, candies, cookies and cakes |  |
| 17 | SPICES, CONDIMENTS, BEVERAGES | Spices (black pepper, salt), condiments (soy sauce, hot sauce), coffee, tea, alcoholic beverages OR *local examples* |  |

**IX. SALT INTAKE**

| **Question#** | **Data Element Label** | **Data Entry Alternatives** | |
| --- | --- | --- | --- |
| 1 | How often do you add chili paste or merken to your food (do not include chili used in cooking)? |  | Never/Rarely |
|  |  |  | 1-2 days/week |
|  |  |  | 3-5 days/week |
|  |  |  | 6-7 days/week |
|  |  |  | Prefer not to answer |
| 2 | How often do you eat fresh chili peppers (include fresh sauces)? |  | Never/Rarely |
|  |  |  | 1-2 days/week |
|  |  |  | 3-5 days/week |
|  |  |  | 6-7 days/week |
|  |  |  | Prefer not to answer |
| 3 | How frequently do you eat salty snacks (e.g., potato chips)? |  | Never/Rarely |
|  |  |  | 1-2 days/week |
|  |  |  | 3-5 days/week |
|  |  |  | 6-7 days/week |
|  |  |  | Prefer not to answer |
| 4 | How often do you eat preserved or salt-cured meats (e.g., ham, bacon, cecina)? |  | Never/Rarely |
|  |  |  | 1-2 days/week |
|  |  |  | 3-5 days/week |
|  |  |  | 6-7 days/week |
|  |  |  | Prefer not to answer |
| 5 | How frequently do you eat fruits of any type? |  | Never/Rarely |
|  |  |  | 1-2 days/week |
|  |  |  | 3-5 days/week |
|  |  |  | 6-7 days/week |
|  |  |  | Prefer not to answer |
| 6 | How frequently do you eat vegetables of any type? |  | Never/Rarely |
|  |  |  | 1-2 days/week |
|  |  |  | 3-5 days/week |
|  |  |  | 6-7 days/week |
|  |  |  | Prefer not to answer |

**X. Medical History**

| **Has a doctor or other health professional ever diagnosed you with:** | | | | |
| --- | --- | --- | --- | --- |
| 1 | Diabetes |  | No | Age of diagnosis ______ years |
|  |  |  | Yes |  |
| 2 | Biliary stones |  | No | Age of diagnosis ______ years |
|  |  |  | Yes |  |
| 3 | If yes, have your gallbladder being removed? |  | No | Age of surgery ______ years |
|  |  |  | Yes |  |
| 4 | Asthma |  | No | Age of diagnosis ______ years |
|  |  |  | Yes |  |

**XI. Dental Health**

| **Question#** | **Data Element Label** | **Data Entry Alternatives** | |
| --- | --- | --- | --- |
| 1 | Do you ever have frequently bad breath or bad taste? |  | No |
|  |  |  | Yes |
|  |  |  | Unknown |
| 2 | Do you ever have swollen, red, tender or bleeding gums? |  | No |
|  |  |  | Yes |
|  |  |  | Unknown |
| 3 | Do you ever saw that gums recede or move away from the tooth? |  | No |
|  |  |  | Yes |
|  |  |  | Unknown |
| 4 | Do you ever have periodontitis? |  | No |
|  |  |  | Yes |
|  |  |  | Unknown |
| 5 | Do you ever have cavity? |  | No |
|  |  |  | Yes |
|  |  |  | Unknown |
| 6 | How frequently do you wash your teeth? |  | Two times/day (or more) |
|  |  |  | One time/day |
|  |  |  | Weekly |
|  |  |  | Never |
|  |  |  | Prefer not to answer |
| 7 | How many natural teeth do you have? |  | N0 natural teeth (0) |
|  |  |  | 1-9 teeth (1) |
|  |  |  | 10-19 teeth (2) |
|  |  |  | 20 teeth or more (3) |
|  |  |  | Prefer not to answer (4) |
| 8 | Do you any removable ddentures? (specify) |  | No (0) |
|  |  |  | Yes, partial (1) |
|  |  |  | Yes, partial (full upper denture) (2) |
|  |  |  | Yes, partial (full lower denture) (3) |
|  |  |  | Yes, complete (full upper and lower) (4) |
|  |  |  | Prefer not to answer (5) |
| 9 | Appearance of oral mucosa |  | Normal |
|  |  |  | A lesion is present |
|  |  |  | Difficult to assess |
| 10 | Evidence of periodontal disease |  | No |
|  |  |  | Yes |

**XII. Interview quality**

| Please rate the interview: |  | Reliable | Comments |
| --- | --- | --- | --- |
|  |  | Difficult to obtain answers |  |
| Interviewer’s code |  | | |
